# Transformative Social and Emotional Learning (T-SEL): The Experiences of Teenagers Participating in Volunteer Club Activities in the Community

**DOI:** 10.3390/ijerph20064976

**Published:** 2023-03-11

**Authors:** Stefan Cojocaru

**Affiliations:** Department of Sociology and Social Work, Alexandru Ioan Cuza University of Iasi, 700506 Iasi, Romania; contact@stefancojocaru.ro

**Keywords:** transformative social and emotional learning (T-SEL), democratic competencies, citizen participation, commitment to social problems, teenager club, UNICEF, Holtis, CASEL

## Abstract

Beginning with social inequities in terms of access to quality, inclusive education for children from disadvantaged backgrounds, especially rural teenagers who leave school early, the Holtis Association, with the support of the UNICEF Representative in Romania, developed a number of interventions intended to facilitate the transition from lower to higher secondary education of students from vulnerable and disadvantaged groups. One of the interventions was the establishment of teenagers’ clubs for volunteer activities, leadership development, and participation in the community to encourage social and emotional learning. (1) Background: This study aims to investigate the extent to which participation in the Holtis club projects contributed to the development of transformative social and emotional learning (T-SEL), as observed from the perspective of the Collaborative for Academic, Social and Emotional Learning (CASEL) competencies among adolescents. (2) Methods: The study was qualitative and used focus groups for data collection. Out of the 65 active clubs, 18 were selected, and their representatives participated in the focus groups. (3) Results: Participating in the club activities, which were organized in the school, with the aim of organizing activities outside the school space stimulated and developed T-SEL competencies among adolescents. (4) Conclusions: The data, which were collected through the voices of teenagers, underlined the personal transformation from the perspective of the CASEL model competencies of SEL, and the study privileged their perspectives.

## 1. Introduction

A number of studies underline the importance of social and emotional learning (SEL) for managing emotions, achieving success, making decisions in a responsible manner, establishing and achieving positive goals, and increasing the degree of empathy and demonstrating empathy towards others, a process that privileges the school environment [1,2,3] and the role of teachers in the process [4,5]. Beginning with a model of the five basic competencies presented by the Collaborative for CASEL [6], Jagers, Rivas-Drake, and Williams analyzed the transformative social and emotional learning (T-SEL) model, which integrates SEL from the perspective of educational equity, meaning “fostering more equitable learning environments and producing equitable outcomes for children and young people furthest from opportunity” [2] (p. 163).

The positive youth development (PYD) theory highlights the important impact of supportive elements in the social environment and individual agency on the well-being of individuals [7]. Lerner launched the Five Cs of the PYD model (2004), through which he stated that there are five indicators: competence, confidence, connection, character, and caring. To these indicators he added a sixth C, contribution, which is the result of the manifestation and mutual strengthening of the other Cs [8]. These indicators participate in developing the well-being and potential of young people and their development as contributors to good families and to community and society [9]. The six indicators highlighted and analyzed by Lerner can also be identified in the competencies presented by the CASEL model.

Beginning in 2019, teenagers’ clubs were developed by the Holtis Association with the support of the UNICEF Representative in Romania as part of the *Quality Inclusive Education* project carried out in Bacau county. The project has several components such as the training of school management teams and teaching staff, including for the development of social–emotional skills, multicultural educational activities, parental education, and youth clubs.

Every month, the clubs organize a project in the community on a theme established within their Organization and Functioning Regulation (Table 1). The have the freedom to design any type of activity that falls within these themes:

Initially, the teenagers’ clubs were developed in secondary schools. In 2021, they were expanded to include high schools with the aim of supporting the students’ transition from secondary school to high school, especially students from rural areas (adolescents aged between 13 and 18). Romania is characterized by an elevated school dropout rate: 2.7% of adolescents who finish the eighth grade no longer attend a form of education (Table 2), and the dropout rates from high school (1.9%) and vocational school (2.7%) affect a large number of adolescents.

In establishing and supporting the clubs (setting up the clubs, running summer schools, providing training for board members, providing grants, etc.), the aim was to support school participation; develop the social and emotional skills of the students; facilitate the transition from secondary school to high school or vocational education; create a context for developing an integrative identity; focus on solving problems; increase community involvement; develop active citizenship; build social contexts for the community recognition of teenagers’ involvement and participation; develop leadership, supportiveness and empathy; build social networks and interactions in new contexts; and so on. The clubs were developed in the school environment with the support of coach-teachers, but the activities are carried out by community members.

In the interval of 2019–2022, 68 clubs for teenagers were developed in Bacau County (Table 3).

Although there has been an increase in the interest towards the integrated development of children and adolescents in current Romanian policies, an interest that concerns the education, welfare, and health sectors, the lack of a concrete approach to the field of formal education with respect to social and emotional skills has also been noted. While the main objective of education is the development of students’ skills and welfare (the assurance of the highest possible level of independence for assisted persons), the objective of the health field is the preservation and promotion of health, with prevention being recognized as one of the most effective and least expensive factors at both societal and individual levels.

Critical thinking, problem solving, perseverance, creativity, emotional health, social skills, ethics, community responsibility, and affection, teamwork, organizational skills, open communication, and solid teacher–student relations are some of the aspects considered important for succeeding in life on all levels, despite the fact that they have been neglected in the design of formal education and are collectively, under the name of non-cognitive skills, considered to be one of the pillars of formal education [11]. The predominant focus of the Romanian education system on the development of cognitive skills—with socio-emotional skills being addressed by a series of electives or left to extra-curricular activities—as well as the lack of continuous training opportunities for orientation towards socio-emotional skills and the lack of resources for teaching staff [12] make the systematic approach of socio-emotional skills difficult. SEL is the concern of certain non-governmental organizations in Romania; however, volunteering is a personal choice, limiting the students’ equal opportunities for complex development [13].

A positive school climate, described as a safe environment in which the student–adult relationship is based on care, is associated with academic performance and with low rates of deviant behavior or delinquency [14]. It is considered, along with social identification, a basic element for improving student performance by acquiring and practicing SEL [4] and for ensuring success in life [15]. The creation of a positive school climate and an increased level of student involvement is also associated with increased academic performance among students [16,17]. However, it must be remembered that the school environment differs between schools, which can lead to a different vision of the school reality as each school’s reality is unique [18]. The creation of a positive school climate that functions as a context to facilitate the students’ integrative development depends on factors such as the manner in which administrative and organizational aspects are handled by the management, the models of interaction promoted between teachers and various other categories of staff or between teachers and students, the fostering of a climate based on transparency, and congruence between what is officially promoted and the behavior of significant figures at the school. These factors are all decisive in the secondary formation of certain attitudes, skills, and knowledge, which are recognized as “parallel education” [19] (p. 7.) The concept of parallel education pays attention to the implicit formation of skills that are not the subject of the curriculum but are instead reinforced in the socialization process of students and teachers in the academic environment [19], which is related to the school culture and organizational climate. From this point of view, the activities in the teenagers’ clubs constitute a form of complementary education in which young people take part in activities in the community as active participants [20], placing special emphasis on T-SEL and aiming to develop the members’ social and emotional skills. This because traditional learning, which is focused on the accumulation of knowledge, does not prepare young people for a civic spirit, integrity, fair governance, problem solving, and teamwork [20].

The concept of “peer culture” emphasizes the influence of relationships between students in school, apart from the influence of narrower peer groups, on their individual results. It reflects, from a relational perspective, the perception of students with respect to the general manner of school relations in which the students engage and their relationship with academic performance, which is reflected at the school level and belongs to the behavioral component of “peer culture”. This distinction is important because they influence and describe, on one hand, the students’ perceptions of relationships and, on the other hand, student behavior. It is also mentioned that “peer culture” is only one component that contributes to the academic performance of students. An important role can also be attributed to the teachers and school principals, who influence the school climate, and to parents [21].

In the view of Lynch, Lerner, and Leventhal, the relational component refers to the manner in which students build an overall picture of the quality and fairness of the school climate and the nature of relationships between colleagues. These factors are important in view of the fact that academic results are associated with school climate and student involvement, which enhance each other [21]. Although “peer culture” is an important component that influences the school climate, there are authors who point out that under certain conditions such as very close friendships between students, “peer culture” may have negative effects on school results, suggesting the need for student involvement in extracurricular activities, team sports, and academic clubs that would ensure the structured supervision of their school activities [22]. Volunteering that is carried out in school could be added to these activities under the supervision of teachers.

Building positive relationships between teachers and student and among students has proven to be beneficial for the latter, as the students become more motivated and tend to become more involved in the learning process, achieving better academic results [23] as they develop skills related to attention, emotional regulation, and coping with challenges [24,25].

## 2. Materials and Methods

### 2.1. Research Objectives

The study aimed to investigate to what extent the participation of members in the projects carried out within the Holtis clubs contributed to the development of transformative social and emotional learning (T-SEL) from the perspective of CASEL competencies among adolescents.

### 2.2. Selection Criteria for the Clubs Taking Part in the Research

Within the project “Technical documentation in order to increase parental education and youth clubs on a national scale”, carried out by the Holtis Association in partnership with the UNICEF Representation in Romania, the establishment of adolescent clubs in secondary schools and high schools aimed to develop leadership and adolescent participation in the community, facilitating the transition from secondary school to high school with the accumulation of new experiences for the development of non-cognitive skills within some extracurricular activities that were supported and supervised by teacher-coaches. At the time of data collection, 65 clubs (rural and urban, from secondary and high schools) were active in Bacău County. Out of these clubs, 18 were selected as models of good practice using the following criteria:

- Perseverance in carrying out projects: Based on this criterion, the clubs that carried out at least one project every month between September 2021 and June 2022 were selected. Based on this criterion, 18 clubs emerged out of a total of 65 active clubs. These 18 clubs were later found to meet the other criteria as well;
- Visibility in the local/international community. Using this criterion, the clubs that were the most visible both in the community and internationally were selected. Visibility refers to clubs that were featured in the local press, clubs that participated in radio broadcasts, and clubs that participated in international events (EYE—European Youth Event);
- Performance in fundraising/resources for carrying out activities (including human resources). This criterion evaluated the ability of clubs to organize projects with the help of community resources through fundraising, donations, etc.;
- Promotion in social media. Based on this criterion, the clubs that were the most active on social media were selected, including those that created Facebook pages, Instagram accounts, etc., and frequently posted the activities they carried out, thus making them much more visible in the community;
- Internal recognition and appreciation. Using this criterion, we selected the clubs which, among the projects they carried out, also carried out team-building activities and promoted the recognition and internal appreciation of the communication and collaboration between the members so that they become as united a team as possible;
- Partnerships between clubs. Based on this criterion, clubs that initiated partnerships with other clubs both inside and outside the community were selected. Partnerships were created between both secondary school and high school clubs, as well as between rural and urban clubs;
- Carrying out projects focused on the needs of the community. Using this criterion, the most active clubs were selected; that is, the clubs that carried out projects focused on the needs discovered at their community level. Projects were carried out to successfully respond to needs identified by club members in their own communities;
- Involvement of teachers. Based on this criterion, the clubs with coaches who were actively involved in the coordination, organization, and implementation of the club’s projects were selected;
- Autonomy of members. This criterion was used to select the clubs that managed, through their members, to organize and coordinate most of the projects carried out without the help of their coach.

### 2.3. Data Collection

This research was qualitative, and data collection was carried out through focus groups attended by members of the clubs. In total, 18 focus groups were conducted, each of them with members of the same club. The variables describing the focus groups are shown in Table 4. We note that the codes describing each group will also be retained when citing statements provided in the interviews.

The focus groups with teenagers were attended by young people who were part of the clubs’ Boards of Directors. The focus groups were organized in workshops that occurred in the period of July–August 2022.

### 2.4. Data Analysis

The focus group discussions were recorded on audiotape to ensure the authenticity of the data extracted from the transcriptions. After transcription, the in vivo codes were selected and categories were constructed. Within the categories, concepts were identified that can be found in the classification made by CASEL; thus, the categorization process underwent a selection process [26], analyzing and framing the concepts for each individual competency. Priority framing was privileged, obviously, although from the point of view of the contents, various points of view of the teenagers could be framed in several competences as they are not isolated and independent from each other. The NVIVO program was used to select the codes and construct the categories.

## 3. Results

The children and adolescents who participated in the research noted many changes since they became members of the Holtis Club (HC). Some of these followed expectations they had when they joined the club, and other changes occurred unexpectedly as a result of the activities carried out and from the way HCs operate as organizations. A number of changes occurred in line with the school atmosphere, where there was a general environment conducive to development. In other cases, good results were obtained regardless of the school environment, and in other cases the club developed through the efforts of its members, at least in the students’ perception, despite what was happening in school. However varied and wide-ranging these results may be, they are eminently positive. Given the qualitative nature of the research, they are at the heart of our research interest.

The changes perceived by the interlocutors following the participation in the club activities were numerous and can be placed on a continuum spanning from a purely individual level—which would concern the individual in the absence of any other person—to a social level—affecting the person in question in relation to others. We speak of a continuum because, in this case, it is difficult to discern where the “individual” ends and the “social” begins. Even when a certain personal quality was discussed—for example, punctuality style—the quality developed while the individual was a member of the club and is assumed to apply to other social contexts as well. These nuances become apparent when the experiences are described in the statements.

### 3.1. Competence in Self-Awareness

The statements below support the idea of group identity, which acquired through participation in group activities. It is an identity that is also supported by the results of the activities, which were usually validated by both students and teachers but also by the community. As belonging to a successful group is an important stage in adolescence and, when associated with success, it contributes to the development and strengthening of optimism as a result of public regard.

*“We are no longer pessimistic.” (…) I mean we always see the glass half full, and we think positive things because you usually attract what you think. We stop thinking about the bad side of the issue. (…) We realize that we need to do more with less.”*

(FG3)

*“Thanks to the club, to the activities, I gradually managed, from one activity to another, to prove myself, and be more positive and associate myself (in public) with the others, to be more integrated, to be part of a group.”*

(FG8)

*“Now we are more optimistic, in my opinion. (…) (That is) Even if a result doesn’t come out (from) the beginning, or it doesn’t work, along the way we get used to it.”*

(FG9)

The idea that socialization reduces anxiety in interacting with others is also emphasized by the statements below, which highlight the importance of group integration through involvement in activities. Here, the club is described as an opportunity for the members to better familiarize themselves with others and develop friendships with schoolmates with whom they would otherwise not talk. In this context, the club becomes a context that facilitates *the development of interpersonal relations between teenagers*, which can contribute to building a more favorable climate for the educational process.

*“Because we socialize a lot and because after you join the club you start to have a lot more confidence in yourself and you start to stop being shy or ashamed of other people, you start to be more open.”*

(FY13)

*“For me, it helps me become more social. Because I’m usually very shy and...” (...) now I can speak more freely.”*

(FY17)

*“It helped me to be more sociable and to step out of my comfort zone more and more. I’m quite a shy person and I try to get out of my comfort zone more and more.”*

(FG8)

*“...and it really helped me to stop being so anxious. I was extremely anxious and shy. At first, because I was (a member?) since the ninth grade, and I didn’t know a lot of people in the high school, but the club really helped me.”*

(FG5)

*“It helped me a lot because I was much more withdrawn and shy. In many of the activities we needed to interact with other people and… (…) I feel it is a transformation.”*

(FG7)

*“... I used to be very shy and didn’t pay attention to anyone and I managed to make friends. For example, I didn’t use to talk to them (interview participants).”*

(FY15)

### 3.2. Competence in Self-Management

The participants’ accounts assert the development of some characteristics that are usually described as temperamental traits, such as extroversion, which is associated with openness and freedom from the fear of expressing one’s own opinion to both peers and some of the teachers, seemingly contributing to the development of closer relationships with teachers, including the coach. However, it is important to also note that this openness is associated with a potential for action in desirable directions, which can be seen as a context for building what is called the territory of “proximal development” or “possible actions”, such as: the capacity to become involved in something, doing something, and validating the ability to relate through concrete actions with tangible results.

*“More handsome, that is we speak out without fear. We’re more venturesome. (More venturesome in relation to the teachers?) Exactly! We really are! (You speak your mind?) Yes, we do! We’re more extroverted.”*

(FG3)

*“And I want to say that it helped me enormously, in the sense that I made friends, I got closer to people, I also got closer to Mrs. D (...) and I managed to let go of a lot of things and get involved in something, to do something.”*

(FY16)

*“Me too, I started to...uh...believe in myself and be confident in myself and cooperate with more people.”*

(FY13)

*“It changed us for the better. We have become much more open with each other and much more creative.”*

(FG4)

*“At first we didn’t really know each other. But now (we know each other and) we are honest with each other and I’m not so shy anymore.”*

(FG4)

*“For myself, I opened up a lot, and with this summer school that we all went to, it seems to me that I opened up a lot and started to communicate and express my point of view. I think that’s very important for anyone, and I think anyone should have the courage to do this.”*

(FG12)

The teenagers we talked to admitted that, upon reflection, they had developed significantly since they became club members and often expressed this evolution with phrases such as *“we have matured”* or *“I take things more seriously”*. Very likely, any person their age would tell us the same thing. The difference in this case is the fact that the process of change defined as “maturation” meant some very concrete things for the teenagers: certain evolutions that can be evaluated and not just vague impressions. Some of the respondents stated that in the clubs, they saw what it meant to be frivolous and adjusted their behavior; others had positive examples to follow. Maturation is both a process—carried out over time, since the subjects joined the club—and a result. In what follows, we prefer to consider maturation a result, because the maturation process would otherwise integrate the entire research. As previously mentioned, the quality of being “mature” or “reliable” involves some indicators referred to by the interviewees to in order to define themselves as such.

“I *have become much more responsible. I didn’t use to be very responsible, I didn’t really get involved, but volunteering helped me to be more responsible, to know that I have something to do and that I have to do that thing, not put it off. I ended up being much more focused on one thing, I no longer have my mind in all directions, that’s why I’m glad that we have an assignment already given to us, because I only focus on that topic.”*

(FG8)

*“I can’t say that before Holtis I was an introverted and unsociable person. On the contrary, I was a jovial, open, sociable person, etc., but Holtis made me realize certain things and I cannot express these things in words because there are several of them, they are more like feelings, because I have different feelings, I look at volunteering with different eyes, better eyes, also the relationship with people. I mean, it made me more patient, honestly. Because before Holtis I was a person who got angry instantly but now I have learned that without patience you can do absolutely nothing and this patience has paid off and now I have learned how to be a more patient person thanks to Holtis, and (I learned) that everything happens in its own time, that is, I don’t have to force a thing: every project in its time, I don’t have to force it to be at that moment because it will come and bear fruit when time deems it necessary.”*

(FY12)

*“I’ve always been quite a social person, but the thing that Holtis helped me the most with would be the matter of patience, because sitting, having to listen to everyone, take their opinions, to listen to all opinions. Anyway, being the chair person, everyone called me with certain problems and all these things, because it was an extra responsibility, it helped me a lot on the patience side, because I learned to have a little more patience, to stop getting angry very quickly and to be a little more zen.”*

(FY12)

During the course of the activities, patience and tolerance also emerged from contact with the reality of life for less fortunate individuals.

*“We are becoming more and more generous and kind. Really more and more. Because we discover cases... (...) in which some people really can’t afford certain things and you try to put yourself in their place. You have a void in your soul and you think: “What would it be like if I were in their place?” And you feel, like, lonely. (...) You learn to be content with what you have. (…) I value more the things I have.”*

(FG1)

The statement below describes the process of change in which the need for collaboration leads to the control and modification of an expression (of “arrogance”), thus leading to a change in the general attitude, which is now carefully managed, and perhaps finally to a complete change of the person (of “character”). However superficial or complete this change may be, the increased capacity for self-control is a sign of maturity and of a more effective integration into the social environment.

*“I get told quite often that I’m arrogant or that I give off an air of arrogance, of... And now ,working with everyone and seeing other personalities, I’ve learned that you may actually appear arrogant, but you’re not, and you know how to you adapt to each person. I think this helped me to change my character to some extent. (…) With facial expressions for example. It very often happened to me to sit and stare blankly and look angry, and be told that I am either upset, or that something bothers me, when in fact there’s nothing wrong, I am simply thinking (...). I even learned how to manage, to control my facial expressions, body language and all that.”*

(FY11)

### 3.3. Competence in Social Awareness

Obviously, maturity and reliability are not only manifested in relationships with others but also in the external environment, where they are more easily noticeable. Another element that describes this process consists, according to the statements of our interlocutors, of overcoming the excessive focus on oneself, an attitude sometimes categorized as “arrogance”, and in an increased availability to resonate with the needs of others, be they club mates, schoolmates, or project beneficiaries. There is now more patience, empathy, tolerance, and flexibility in the relationship with oneself and with others, which came as a result of a personal assessment and transformation.

*“Beforehand I didn’t really pay attention to what others were saying, but now I talk, I socialize more.”*

(FG1)

*“Well, it made me more sensitive. I’m already sensitive, but it made me see situations somewhat, to help people. (…) And I like to be like that, I like to have that “empathy” for the person next to me, because it makes me a better person and see the situation differently.”*

(FG6)

*“Well, I expected people to be happy, and I was happy too, together with them. (What did that project help you do?) Well, I became kinder.”*

(FG10)

*“I can be better with people.”*

(FG10)

*“It changed in the way that I used to be meaner with my colleagues. (…) worse, I ignored them... (…) So, now I no longer ignore them, I talk to them, I make people laugh… ”*

(FG17)

### 3.4. Competence in Relationship Skills

Somewhat predictably, since this is a group in which the members must work together and cooperate, the top-ranked finding in terms of the frequency of occurrence in the children’s statements was that participating in the club made them more sociable, helped them more easily accept the presence of other people and enter, as a first step, a conversation with them. In a way, participating in the club’s activities helped them step out of themselves and fully enter the social world. An initial understanding of the transformation for the better of the club members would be through the explanation offered by them that, simply, they had to speak, argue, and support their own ideas once they joined the club. Once they settled into conversation with their colleagues, they dared to express more. Beginning to speak more freely was noted by participants from several perspectives, among which personal evolution in terms of the ability to interact with others and to listen was highlighted as the foundation for the construction of social relationships that can protect one from loneliness by making new friends when one joins other social structures.

*“For me (the club) is a way to evolve, to get out of... I’m not very sociable, it’s an opportunity to express myself better and feel more comfortable around people.”*

(FG5)

*“Once you join such a club you are willing to communicate because you have to give your opinion on projects.”*

(FG5)

*“Before, I didn’t really talk to people on the street, to strangers. I didn’t really pay attention to what others said, but now I talk, I socialize more.”*

(FG1)

*“And we learned to talk to more people, that’s how we developed.”*

(FY15)

*“I didn’t use to socialize much. I used to socialize little and now I do it more and more.”*

(FG1)

Likewise, the ability to speak in public is the result of beginning to speak more freely. This was explained as being based on courage and supported by the positive pressure of the group, which values expression in general and provides feedback that can be interpreted in terms of personal traits such as being seen as “more fun, funnier, friendlier”, traits which may be related to social validation.

*“You have more courage to speak in public, you have the opportunity to overcome the fear of speaking in public especially.”*

(FG2)

*“I see myself as more fun, funnier, friendlier.”*

(FY17)

*“Plus I’m better at talking to people now, not that I had a problem with that before, but I was more... I used to ask someone else to speak on my behalf, now I don’t have that problem anymore.”*

(FY11)

The very attitude towards communication with others changed: before joining the club, one interlocutor was of the opinion that speech was powerless. After joining, they found out that their influence as a person increased significantly due to what they say and, obviously, do, lending meaning to their own contributions in their relations with others and contributing to the development of their ability to talk with “the people around”.

*“Before I joined this club, I used to avoid socialising. I didn’t like talking to people because it seemed pointless to me. At one point or another, everyone leaves and it makes no sense to help them with anything. But when I joined the club, I saw that it wasn’t like that at all, and that every person was happy when they received attention, no matter how little. Now it’s much easier for me to talk to children, to the people around.”*

(FG7)

The fact that participating in club activities represented a context for discovering and developing existing skills was also emphasized by the participants’ statements regarding leadership activity, supporting the idea that practicing a skill can be associated with better self-knowledge, and that the creation of such contexts can be very important in teenage years. Practicing leadership skills was described as being accompanied by increased confidence in one’s own strengths, a kind of self-validation and improvement of one’s abilities alongside group members, as reported in the statement below:

*“It made me much more confident, I’ve always been told that I have leadership potential, to support people, to advise them, to engage them, but I never trusted myself. It was... I always wanted to be a leader, but I didn’t have the confidence that I could be, even if I always aimed for perfectionism, I didn’t think that I was even capable of achieving it, but every time and together with my team I realized that I could do anything I set my mind to if I really wanted to, and it helped me correct my flaws and appreciate my qualities more.”*

(FY16)

### 3.5. Competence in Responsible Decision Making

Participating in the club activities, in projects carried out jointly by teenagers, represents an extremely powerful socialization and behavior-modeling exercise. At the same time, these mechanisms of involving adolescents in extracurricular activities based on voluntary participation develop skills that are then transferred to activities specific to the classroom and to the formal learning process.

*“My parents, the teachers and so on, think that I’ve changed, because before this I was very naughty, I wasn’t paying attention in class, I did naughty things. Now I’ve changed. I’m a little more attentive in class, I take notes.”*

(FY17)

The future, especially for younger secondary school children, does not only mean a career or a profession but also the Holtis Club. Among the criteria for choosing a high school was the existence of such a club; at least one high school became aware of this “trend” and included the club in its educational offer. Additionally, some older high school students raised the issue of continuing their presence, in one way or another, in the Holtis Club. It is interesting that the Holtis Club, which was active in high schools, was among the educational offers presented by the high schools in Bacău; the existence of a club it began to be a point of attraction for secondary school teenagers who were about to make the transition to high school.

*“I want to be a Holtis member in high school as well. (Does this mean that this is a criterion for choosing your high school? Will you choose a high school that has Club Holtis or would you contribute to the establishment of a club?) More like this, I think (the latter option).”*

(FG4)

*“I want us to get into a high school with a Holtis club.”*

(FY15)

*“I would like to focus on something, teamwork. I would look for a high school that is similar to the club.”*

(FG9)

The simplest decision-making procedure is the one in which the best option is chosen when there are several proposals—and most of the time there are, because that is how the club works. The option is chosen by vote or consensus.

*“Usually everyone gives their opinion and we see which one is the best.”*

(FY15)

*“Everyone had their say and we voted.”*

(FG9)

*“It seems to me that everything comes naturally and in abundance, ideas come from all sides, proposals from all sides, we choose the best ones and put them into practice. We are realistic...”*

(FG7)

*“There are several proposals from the members, we meet, we discuss each one and which is the most voted... If there are more children who like my idea, that one is chosen.”*

(FG2)

## 4. Discussion

### 4.1. T-SEL in the Teenagers’ Club

The model of SEL competencies developed by CASEL and refined in the model presented and analyzed by Jagers, Rivas-Drake, and Williams [2] represents an extremely useful grid for analyzing the data obtained from adolescents in the focus group. In the Tentative List of Relevant Concepts/Constructs for Forms of Social and Emotional Learning, one can find the five competencies, which are analyzed from T-SEL perspectives: self-awareness, self-management, social awareness, relationship skills, and responsible decision making [2] (p. 166). The teenagers’ club, which was aimed at practicing and developing social and emotional skills, plays an important role in the development of adolescents as a social space for interaction and a creator of new action contexts. Participating in club activities, along with exercising autonomy and leadership, also contributes to achieving academic success: teenage members improved their school results and the level of their aspirations. The influence of SEL in improving school results was also emphasized by other authors [1,15,20] and is reflected in the teenagers’ statements with respect to improved attention in class, grades obtained, the courage to speak freely, etc. Youth activism is considered by Fredericks [27] to be a participatory form through which SELs are actualized; the activism of young people in the community represents opportunities to develop their skills in contexts that are different from the usual school environment through action in the community [27]. The connection between education and action is a key element for social change [28] Adolescents are becoming increasingly civically active, taking on responsibilities as participants and even initiators of community actions. The data obtained can be found in the transformative learning analysis carried out by Mezirow through which those fixed assumptions and expectations became more inclusive, open, and reflective [29]. The composition of the clubs was heterogeneous (adolescents aged between 13 and 18 of different ethnicities, religions, economic situations, social status, and school situations). This contributed to increasing the assurance of equity, as this study is also concerned with providing and creating contexts in which adolescents participate in “actions that attempt to resist, disrupt, and dismantle the inequities perpetuated by dominant culture that keeps their neighbour in an oppressed, marginalized position” [30]. This is achieved by encouraging the organization and participation of club actions in the community so that the exposure of young people is as wide as possible and is achieved in a real social context. The importance of these non-academic forms of education have been emphasized by various authors, who emphasize character education [31], moral education [32], forms of preventing various risk behaviors [33], and the integration of feelings for the fulfilment of tasks [34]. The perspective of CASEL competencies in the context of T-SEL seems to have a wider applicability as it is useful not only for understanding various components and dimensions of the five competencies but also for guiding interventions both among teachers [5,35] and in the community [36,37].

The activities carried out by young people in the community, based on plans and projects that were proposed, analyzed, and carried out by the youth through debates, visibly participate in the development of SEL because as Cicchetti and Rogosch also state, SEL develops in a social context [38]. The club is a social space and is complementary to the school space through which social contexts of interaction are generated and multiplied.

By participating in the conception, design, and realization of these activities, the teenagers became more optimistic, had a positive attitude, and were more motivated to develop as they were stimulated by the construction of an identity of belonging to the club and by confidence in their own strengths (*self-awareness competence*). The analysis of the codes selected in the analysis demonstrated that confidence in one’s own power, courage, and optimism is among the most common statements from the teenagers who participated in the focus groups.

Exploring one’s own abilities to solve problems contributes to the maturation process, to assuming responsibilities for solving problems [39], to the willingness to listen, and to self-manage (*self-management competence*), results that are also in agreement with other studies [40]. Volunteering is also animated by a feeling of generosity towards others, sensitivity towards the needs of others, and the desire to support fellow human beings. There were other qualities that the teenagers stated were the result of their involvement in the activities carried out by the club members. The desire to help one’s peers, accompanied by social recognition, developed the teenagers’ sense of belonging to the club and the community; the activities carried out every month provided the teenagers with a diversity of interactions, which were marked by a special appreciation by them (*social awareness competence*).

Sociability, accentuated and enhanced by immersion in the real world and by the ability to interact with others in a collaborative process focused on tasks and problem solving, contributes to the development of *relationship skills competence* (engagement, sharing, helping, leadership, and multicultural competence).

The decision-making process for the collective good was identified in the teenagers’ statements, and the projects carried out by the teenagers in the community contributed to providing the grounds for pluralism, distributive justice, and collective well-being (*responsible decision making competence*).

### 4.2. Future Research Directions

This study, as part of the evaluation process for the *Quality Inclusive Education* project, had an exploratory purpose: to identify to what extent the club, as a learning environment complementary to the school, participated in the development of T-SEL. It appears from the analysis of the data provided by the teenagers in the focus groups, all five T-SEL competencies were identified as a result of the involvement in the club activities. For the future, it would be extremely useful to apply tools to measure these skills among all members of these clubs as there are currently over 1300 teenagers who are active members in the 68 clubs. At the same time, the tools for measuring these skills could be used to identify the differences between secondary school and high school club members. Another direction of research would be to study how participation in club activities influenced the careers of those completed high school (there are already high school graduates who were members of the club during their teenage years).

### 4.3. Limits

The present qualitative research is an incursion into the universe of young people and their subjective ways of describing personal experiences within the volunteering activities organized by clubs. At the same time, the study is not a grounded-theory type because it did not set out to develop new orientations and theoretical approaches, but instead to check if the competencies from the CASEL model can be identified in the statements of the young participants in the study. Although there are several theoretical models to which the collected data could be reported (for example, positive young development pr the theory of resilience) we considered the CASEL model useful for this research.

## 5. Conclusions

The participation of teenagers in the activities of their clubs represents a learning framework that is complementary to the school environment, which participates in the development of T-SEL. The club is a socialization framework through which new social contexts are created in the social reality, supporting the development of these skills. The multiple diverse experiences in the community contribute to the development of a positive attitude towards one’s own person and towards others, to well-being, to maturation and assuming responsibility, to leadership, to generosity, and to the development and crystallization of an identity capable of contributing to social justice and collective well-being. The challenges of today’s society, marked by rapid and profound changes, contribute to the deepening of uncertainty, which is perceived differently by each of us. The teenagers’ membership in a club and their active participation is a support for managing this uncertainty as a factor of stability was built into the peer group. The development of T-SEL within a non-formal learning process that was adapted to the conditions of the real world, caused the adolescents to become more connected to the community, sensitive to the needs of others, and to adapt to the circumstances of society, which is in a permanent and fast state of evolution. All five T-SEL competencies were identified in the statements of the focus group participants as a result of participating in the club activities. The results obtained within the presented clubs were an additional motivation for the continuation in 2022 of scaling up and establishing new clubs nationwide. Thus, at the end of 2022, 141 adolescent clubs were operating in secondary schools and high schools in rural and urban areas.

## Figures and Tables

**Table 1:** Monthly themes for the club projects.

Month	Theme
September	Education And Innovation Month
October	Community Month
November	Tolerance Month
December	Generosity Month
January	Healthy Living Month
February	Love And Dance Month
March	Vocation and Career Orientation Month
April	Environment Month
May	Family Month
June	Sports Month

Note: July and August are school holiday months.

**Table 2:** The evolution of transition rates and of school dropout rates for high school and vocational school education (2010–2020).

Indicators	2010/2011	2011/2012	2012/2013	2013/2014	2014/2015	2015/2016	2016/2017	2017/2018	2018/2019	2019/2020
Rate of transition rate to high school and vocational school	-	96.7	96.7	94.4	93.9	93.7	94.9	94.3	95.3	97.3
Dropout rate in high school education	3.2	3.8	2.8	2.8	3.7	3.5	2.5	2.5	2.5	1.9
Dropout rate in vocational education	19.8	30.4	7.9	4.3	5.0	4.2	3.5	3.9	3.8	2.7

Source: National Institute of Statistics; Ministry of Education [10].

**Table 3:** Active clubs in the Bacau County.

Educational Level	Number	Location	Number
Secondary school	47	Rural	38
High school	21	Urban	30
Total	68	Total	68

Source: www.qie.ro (accessed on 14 December 2022).

**Table 4:** Characteristics of focus group participants.

Code of Focus Group	Club Residence	Level of School
FG01	Urban	Secondary school
FG02	Urban	High school
FG03	Urban	High school
FG04	Rural	Secondary school
FG05	Urban	High school
FG06	Urban	High school
FG07	Urban	High school
FG08	Urban	High school
FG09	Rural	Secondary school
FG10	Rural	Secondary school
FG11	Urban	High school
FG12	Urban	High school
FG13	Rural	Secondary school
FG14	Urban	Secondary school
FG15	Rural	Secondary school
FG16	Urban	High school
FG17	Rural	Secondary school
FG18	Rural	Secondary school

## Data Availability

The authors declare that data or models are not deposited in an official repository.
